# Cefmetazole for bacteremia caused by ESBL-producing enterobacteriaceae comparing with carbapenems

**DOI:** 10.1186/s12879-016-1770-1

**Published:** 2016-08-18

**Authors:** Takahiko Fukuchi, Kentaro Iwata, Saori Kobayashi, Tatsuya Nakamura, Goh Ohji

**Affiliations:** 1 Division of Infectious Diseases Therapeutics, Kobe University Graduate School of Medicine, 7-5-2 Kusunokicho, Chuoku, Kobe, Hyogo 650-0017 Japan; 2 Department of Microbiology and Infectious Disease, Kobe University Graduate School of Medicine, 7-5-2 Kusunokicho, Chuoku, Kobe, Hyogo 650-0017 Japan; 3 Department of Clinical Laboratory, Kobe University Hospital, 7-5-2 Kusunokicho, Chuoku, Kobe, Hyogo 650-0017 Japan

**Keywords:** ESBL, Extended spectrum beta-lactamase, Bacteremia, Cephamycin, Cefmetazole

## Abstract

**Background:**

ESBL (Extended spectrum beta-lactamase) producing enterobacteriaceae are challenging organisms with little treatment options. Carbapenems are frequently used, but the emergence of carbapenem resistant enterobacteriaceae is a concerning issue, which may hinder the use of carbapenems. Although cephamycins such as cefoxitin, cefmetazole or cefotetan are effective against ESBL-producers *in vitro*, there are few clinical data demonstrating effects against bacteremia caused by these organisms.

**Methods:**

We performed a retrospective observational study on cases of bacteremia caused by ESBL-producers to investigate the efficacy of cefmetazole compared with carbapenems. We also evaluated whether the trend of antibiotic choice changed over years.

**Results:**

Sixty-nine patients (male 34, age 69.2 ± 14.4), including two relapse cases, were reviewed for this analysis. The most common causative organisms were *Escherichia coli* (64, 93 %), followed by *Klebsiella pneumoniae* and *K. oxytoca* (2 each, 4 %). The group that received carbapenem therapy (43, 62 %) had increased severity in the Pittsburgh Bacteremic score than the group that received cefmetazole therapy, (1.5 ± 1.5 vs 2.5 ± 2.1, *p* = 0.048), while analysis of other factors didn’t reveal any statistical differences. Five patients in the carbapenem group and one patient in the cefmetazole group died during the observation period (*p* = 0.24). CTX-M-9 were predominant in this series (59 %). Infectious disease physicians initially recommended carbapenems at the beginning of the current research period, which gradually changed over time favoring the use of cefmetazole instead (*p* = 0.002).

**Conclusion:**

Cefmetazole may be safely given to patients with bacteremia caused by ESBL-producers as a definitive therapy, if one can select out relatively stable patients.

## Background

Spreading antibiotic resistance is a global concern. In terms of enterobacteriaceae, extended spectrum beta lactamase (ESBL) producing enterobacteriaceae and carbapenem resistant enterobacteriaceae (CRE) are the most concerning pathogens [1]. Infections caused by ESBL producing enterobacteriaceae (ESBL-E) are associated with higher mortality [2] and medical cost [3–5].

There is a strong relationship between antibiotic use and increase in prevalence of resistant pathogens. The drugs most often used against ESBL-producers are carbapenems [6]. Research has elucidated that recent exposure (3 months prior) to antibiotics was a parameter consistently associated with detection of CRE [7, 8]. Overuse of carbapenems and subsequent selective pressure can contribute to the spread of CRE. CREs are rarely identified in Japan as of this writing, but overreliance on carbapenems may induce CREs. Thus, CRE became an emerging pathogen, especially in Europe and North America.

Cephamycins, such as cefmetazole and flomoxef have good activity against ESBL-producers *in vitro.* However, clinical data regarding the potential value of cephamycins for the treatment of ESBL-associated infections are scarce [9]. Some are also reluctant to use cephamycins as first-line therapy for ESBL-producing organisms [10].

We therefore conducted a study to evaluate the efficacy of cefmetazole against bacteremia caused by ESBL-E. In addition, the trend of the prescription of antibiotics against ESBL-E bacteremia were evaluated over years to see the effectiveness of Infectious Disease consultants in avoiding the overuse of carbapenems.

## Methods

We conducted a retrospective observational study at a 930-bed university hospital in Kobe, Japan. All patients admitted from 1st January 2008 through until 31 December 2013 who had ESBL-E isolated from blood cultures were identified. The clinical records of all 125 who had positive blood cultures of ESBL-E, and those who were treated with carbapenems or cefmetazole as definitive therapy were reviewed. ESBL-E were defined as positive strains of the double disc synergy test [11].

Duplicated cultures, pediatric patients, those judged as contamination by the infectious disease team, and palliative care patients were excluded.

The following data was collected from the clinical records; age, gender, community acquired or nosocomial infection, origin of bacteremia, causative pathogens, comorbidities (using Charlson comorbidity scoring index; myocardial infarction, congestive heart failure, peripheral vascular disease, dementia, chronic pulmonary disease, connective tissue disease, peptic ulcer disease, liver disease, diabetes mellitus, hemiplesia, renal disease, tumor or lymphoma with or without metastasis, leukemia, and AIDS), vital signs (using Pittsburgh bacteremic Score and SOFA score), admission into ICU, bacterial profile (single pathogen or mixed infections, resistance against cefmetazole, resistance against fluoroquinolones), treatment profile (empirical treatment, definitive treatment, consultation with infectious disease physicians, the efficacy of empirical antibiotics).

Primary outcomes were defined as a death within 30 days after the documentation of bacteremia and a relapse of bacteremia caused by the same pathogen.

In addition, we evaluated the relationship between treatment options and consultation with infectious disease physicians over years. Furthermore, we estimated the resistant genes of ESBL-E. Genetic detection and genotyping of TEM, SHV, and CTX-M were performed by using PCR with bacterial DNA, which was extracted from the isolates by boiling the bacterial suspensions. A solution with an extracted DNA concentration of 0.1 ng/mL was used as the template for PCR analysis. In the case of genotyping of CTX-M genes, 4 primer sets that amplify group-specific CTX-M genes were used, as described previously: the CTX-M1 group includes CTX-M-1, CTX-M-3, CTX-M-10 to CTX-M-12, CTX-M-15, CTX-M-22, CTX-M-23, and CTX-M-28 to CTX-M-30; the CTX-M2 group, CTX-M-2, CTX-M-4 to CTX-M-7, CTX-M-20, and Toho-1; the CTX-M8 group, CTX-M-8; and the CTX-M9 group, CTX-M-9, CTX-M-13, CTX-M-14, CTX-M-16 to CTX-M-19, CTX-M-21, CTX-M-27, and Toho-2. The PCR products were analyzed using 2 % agarose gel electrophoresis and visualized by staining with ethidium bromide.

The statistical analysis was performed using STATA 13 (STATA corp. LP, College Station, Texas, USA). The Mann-Whitney test was used to compare the two groups, also an extension to the Wilcoxon rank-sum test, the ‘nptrend’ STATA command, was used to assess trends in proportions across ordered groups. All test were two-tailed and *p* values ≤ 0.05 were used for statistical significance testing.

## Result

**Fig. 1:**
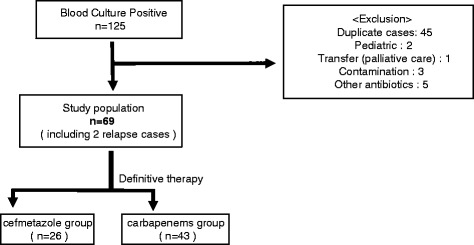
Enrollment of the patients

Sixty-nine patients with ESBL-E bacteremia were reviewed for the analysis (Fig. 1). Twenty-six (38 %) patients were given cefmetazole (CMZ) as definitive therapy, while 43 patients (62 %) were given carbapenems (CPs), of which meropenem was used most (39), followed by doripenem (3), and biapenem (1). The average age of the patients was 67.5 ± 14.6 and 71.2 ± 13.0 in CMZ group and CPs group, respectively (Table 1, *P* = 0.18). SOFA score tended to be higher in CPs group, although it did not reach statistical significance (*P* = 0.055). The Pittsburgh Bacteremic Score in the CPs group was significantly higher than CMZ group (*P* = 0.048). The most frequent origin of infection was the urinary tract in both groups (CMZ group: 18, 69 %, CPs group: 17, 40 %), followed by hepatobiliary infections (CMZ group: 5, 20 %, CPs group: 8, 19 %). The most frequently isolated pathogens were *Escherichia coli* in both groups (CMZ group: 25, 96 %, CPs group: 39, 91 %). The CMZ group had one case caused by *Klebsiella pneumonia*e, and the CPs group had two *K. oxytoca* and one *K. penumoniae.* The most used empiric therapies in the CPs group were carbapenems (27; meropenem 21, doripenem 3, biapenem 2, imipenem/cilastatin 1), followed by cefazolin (5), ceftriaxone (4), sulbactam/cefoperazone (3) (Table 2). The most used empiric therapies in the CMZ group were meropenem (6) followed by cefepime (4), tazobactam/piperacillin (4), sulbactam/ampicillin (3), ceftriaxone (3), cefmetazole (2). Antibiotic coverage rates are shown in Table 1. Genetic analysis revealed CTX-M-9 was predominant in isolated strains in this series (39, 59 %), followed by CTX-M-1 (21, 32 %), M2, TEM/SHV, KOXY (2 each, 3 %).

**Table 1:** The Baseline Characteristics of the Patients

		Cefmetazole group	Carbapenems group	*p* value
	number	26	43	-
	male	12	22	-
	age	67.5	71.2	-
Source	UTI	18 (69 %)	17 (40 %)	-
Pathogen	*E.coli*	25 (96 %)	39 (91 %)	-
Outcome	survive without relapse	25	36	0.243
	death	1	5	
	relapse	0	2	
Patient profile	SOFA	2.8 + -2.8	5.0 + -4.5	0.055
	Charlson	7.1 + -3.4	6.8 + -2.8	0.85
	Pitt. Bact. Score	1.5 + -1.5	2.5 + -2.1	0.048
	admission to ICU	5 (24 %)	17 (39 %)	0.111
	community acquired	10 (38 %)	11(26 %)	0.29
Bacterial profile	mixed infection	2 (8 %)	7 (16 %)	0.466
	cefmetazole:R	0 (0 %)	1 (2 %)	1
	fluoroquinolone:R	19 (73 %)	34 (79 %)	1
Treatment profile	ID consult	19 (73 %)	21 (49 %)	0.077
	empiric cover	13 (50 %)	27 (63 %)	0.325

**Table 2:** Empiric therapies and proportion of unfavorable outcomes on both groups of cefmetazole (CMZ) and carbapenems (CPs)

CMZ group				CPs group			
	Empiric therapy	Number	Unfavorable outcomes		Empiric therapy	Number	Unfavorable outcomes
Empiric therapy effective against the causative organisms	CPs	7	1:died	Empiric therapy effective against the causative organisms	CPs	27	3:died, 2:relapsed
	tazobactam/piperacillin	4	none				
	CMZ	2					
Empiric therapy ineffective against the causative organisms	cefepime	4	none	Empiric therapy ineffective against the causative organisms	cefazolin	5	1:died
	ceftriaxone	3			ceftriaxone	4	1:died
	sulbactam/ampicillin	3			sulbactam/cefoperazone	3	none
	others	3			Others	2	

Six patients died during the study period (CMZ group: 1, CPs group: 5), while 2 patients in the CPs group relapsed (became bacteraemic) after treatment with meropenem. As a result, 96 % of patients in the CMZ group and 84 % of patients in the CPs group survived without relapse (*p* = 0.24).

The CPs group and the CMZ group had resistant genes such as CTX-M9 (20, 16), CTX-M1 (13, 7), CTX-M2 (1, 1), KOXY (2, 0), TEM/SHV (2, 0), respectively. Five patients in the CPs group died, and their ESBLs were CTX-M9 (1), CTX-M1 (3), KOXY (1).

Resistance against cefmetazole was rare (only 1 case in CPs group, or 1 %).

The consultation to infectious disease physicians tended to be higher in the CMZ group, (75 % vs 49 %, *p* = 0.077).

**Fig. 2:**
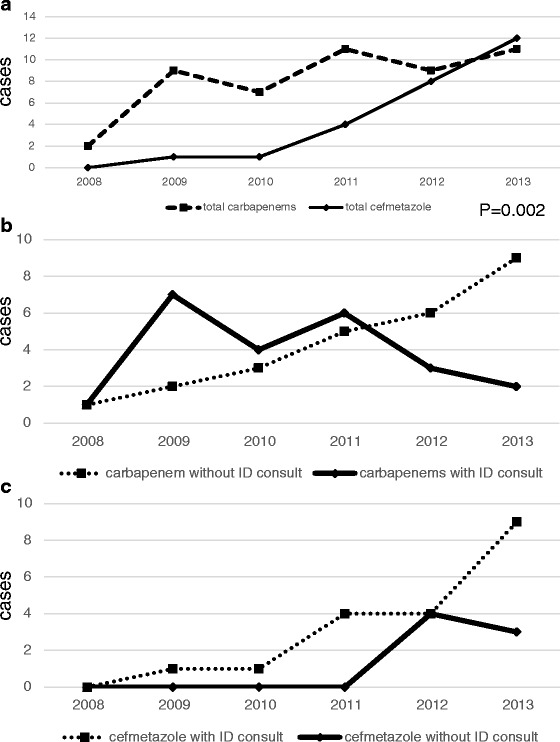
**a** use of study antimicrobials by year. **b** The number of the patients using carbapenems with or without ID consultation. **c** The number of the patients using cefmetazole with or without ID consultation

The selection of antibiotics, ID consultation, and outcomes of each-year are shown in Fig. 2. The CMZ group and the CPs group had a significant difference in trend over years with the use of CMZ becoming much more popular recently (*p* = 0.02).

## Discussion

This observational study demonstrated that bacteremia caused by ESBL-E were effectively treated with cefmetazole as definitive therapy, and this suggests cefmetazole can be a candidate as an alternative for carbapenems. The patients in both groups had low mortality. Of note, relapse of bacteremia caused by ESBL-producer occurred only in the carbapenems group, although these relapsed patients were originally treated with sufficient quantity and duration of carbapenems.

Over years both cases with and without Infectious Diseases physician consults started to use CMZ as the definitive therapy probably due to confidence in the use of CMZ against ESBL-E (Fig. 2).

The rationale of antimicrobial therapy against sepsis or bacteremia is as follows. As empirical therapy, broad-spectrum antibiotics must be selected with one or more agents active against likely bacterial pathogens after obtaining appropriate cultures. It should be reevaluated according to the results of cultures to optimize efficacy, to prevent resistance, to avoid toxicity, and to minimize costs [12]. When treating ESBL-producers, carbapenems have unnecessary coverage against non-fermenters such as *Pseudomonas* and *Acinetobacter, *which would make carbapenems unfavorable antibiotics.

Cefmetazole has a narrower spectrum compared to carbapenems and does not cover *Pseudomonas* or *Acinetobacter.* These characteristics would provide another advantage of cefmetazole over carbapenems. Cefmetazole can play an important role in antimicrobial stewardship programs.

Studies have shown the efficacy of cefmetazole against ESBL producing enterobacteriaceae. Matsumura et al. found that treatment with cefmetazole or flomoxef against bacteremia caused by ESBL-producing *E. coli* were similarly effective compared with treatment with carbapenems in a retrospective multicenter study using a propensity score-adjusted analysis [13]. This study demonstrated the efficacy of two cephamycins against ESBL-producing *E.coli* in both empirical and definitive therapy cohorts. However, use of cefmetazole as an empirical therapeutic agent is not realistic, because one cannot predict whether the causative pathogens were really ESBL-E prior to the culture result. Therefore, we rather tried to focus on definitive therapy alone, where cefmetazole could be utilized better.

Doi A et al. showed the efficacy of cefmetazole against pyelonephritis caused by ESBL-producing enterobacteriaceae [14]. Bacteremic patients were not included in the CMZ group in this study (0/7), as opposed to carbapenems (8/12). Our study focused on bacteremic patients, which makes our results applicable in a wider setting.

Considering other candidates, there are various studies claiming the efficacy of beta-lactam/beta-lactamase inhibitor (BLBLI) against ESBL-producers, but with some controversies. Piperacillin/tazobactam is a promising antibiotic, although piperacillin has unnecessary coverage against *Pseudomonas*. In addition, the role of piperacillin/tazobactam for patients with ESBL-E remains unclear. A meta-analysis described BLBLI as being non inferior compared to carbapenems [2]. However, some research in the US and Taiwan and a cohort study in the US showed higher mortality when piperacillin/tazobactam is used when compared with carbapenems [6, 15, 16].

On the other hand, ertapenem has good activity against ESBL producers, and has the advantage of not covering *Pseudomonas,* a different characteristic from other carbapenems [17]. Ertapenem is still much broader than CMZ, making CMZ more advantageous over ertapenem. Ertapenem use led to the selection of multi-drug resistant bacteria at a Singapore hospital [18].

By the same token, cefepime could be effective against the strains in North America. In East Asia, however, it has less activity against ESBL-E (78.6 %) [19]. Similarly, aminoglycosides keep their activity against ESBL-E. Despite this, many physicians are reluctant to use aminoglycosides for fear of adverse effects such as nephrotoxicity [20].

Regarding the diversity of ESBL-E, there are at least 300 strains in the world. Taiwanese research revealed high resistance of ESBL-E against CMZ (40 %), but most were CTX-M-14 or CTX-M-15 [21]. In our study, CTX-M-9 was the most common strain (51 %).

There are several limitations in this study. First, this is only a retrospective observational study at one institution. Resistance patterns differ among hospitals, regions, or countries. Our results might not be applied to other settings, particularly where the genotype of ESBL producers are different. However, settings with similar resistant patterns to our hospital may use CMZ like our practice. Secondly, our patients had relatively less severe infection than those in the previous studies. Third, selection bias could not be completely removed in both groups.

Even though we are aware of these limitations, the fact that most patients with bacteremia caused by ESBL-producers were successfully treated with cefmetazole was quite promising. For clinically stable patients, who are already improving on empirical antimicrobial treatment, or without clinical deterioration can be a good candidate for de-escalation to cefmetazole with close clinical monitoring. Prospective studies in various settings may confirm our findings in the future.

## Conclusion

Our study suggests cefmetazole may be effective against bacteremia due to ESBL-producing Enterobacteriaceae as a definitive therapy, therefore allowing the sparing of carbapenems against the organisms. Further research, such as prospective interventional studies are needed to confirm our findings.

## Abbreviations

CMZ: Cefmetazole
CPs: Carbapenems
CRE: Carbapenems resistant enterobacteriaceae
ESBL: Extended spectrum beta-lactamase
